# The Proprotein Convertase BLI‐4 Is Required for Axenic Dietary Restriction Mediated Longevity in *Caenorhabditis elegans*

**DOI:** 10.1111/acel.70058

**Published:** 2025-04-08

**Authors:** Ping Wu, Lieselot Vandemeulebroucke, Huaihan Cai, Bart P. Braeckman

**Affiliations:** ^1^ Laboratory of Aging Physiology and Molecular Evolution, Department of Biology Ghent University Ghent Belgium; ^2^ Overseas Pharmaceuticals, Ltd. Huangpu District Guangzhou China

**Keywords:** axenic dietary restriction (ADR), BLI‐4, *Caenorhabditis elegans*, longevity, neuropeptide signaling, proprotein convertase, proteomics

## Abstract

Dietary restriction (DR) is a well‐established method for extending lifespan across various species, including *C. elegans* . Among the different DR regimens, axenic dietary restriction (ADR), in which worms are grown in a nutrient‐rich sterile liquid medium, yields the most powerful lifespan extension. However, the molecular mechanisms underlying this longevity phenotype remain largely unexplored. Through a pilot screen of candidate genes, we identified the proprotein convertase BLI‐4 as a crucial factor in neurons for modulating lifespan under ADR conditions. BLI‐4's role appears to be specific to ADR, as it does not significantly impact longevity under other DR regimens. We further explored the involvement of different *bli‐4* isoforms and found that isoforms *b, f, i* and *j* redundantly contribute to the ADR‐mediated lifespan extension, while the *bli‐4d* isoform is mainly involved in development. Proteomics analysis revealed that the loss of BLI‐4 function under ADR conditions specifically downregulates GOLG‐2, involved in Golgi complex organization. This gene also partially mediates the longevity effects of BLI‐4 under ADR conditions. Our findings highlight the importance of neuronal BLI‐4 and its downstream targets in regulating lifespan extension induced by ADR in *C. elegans* .

## Graphical abstract

In ADR‐induced longevity in *C. elegans* , neuronal BLI‐4 processes neuropeptides, which are then released with the assistance of UNC‐31 and GOLG‐2.

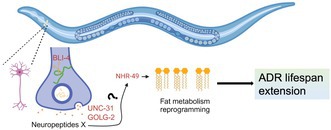

## 1 Introduction

Dietary restriction (DR), lowering food intake without causing malnutrition, is the most robust and consistent non‐genetic intervention known to improve lifespan and healthspan across a spectrum of organisms ranging from single‐celled yeast to primates (Fontana et al. 2010). In the nematode *Caenorhabditis elegans* , a broad range of DR regimes have been identified, such as bacterial dilution, food deprivation, and *eat* mutation, which all extend lifespan to varying degrees. Axenic dietary restriction (ADR) represents a unique DR regimen that induces the most robust lifespan extension. The longevity pathways associated with ADR are independent of most key factors that are known to be involved in longevity mediated by other DR regimens (e.g., DAF‐16/FoxO, SKN‐1/Nrf2, HIF‐1, HSF‐1, PHA‐4/FoxA and TOR) (Bishop and Guarente 2007; Castelein et al. 2014; Greer et al. 2007; Hansen et al. 2007; Panowski et al. 2007). In ADR, worms are grown in a nutrient‐rich sterile liquid medium and exhibit phenotypic features that resemble those of dietary restricted animals, including slenderness, delayed development, reduced reproductive output, and lifespan extension (Houthoofd et al. 2002). Despite the strong lifespan extension observed in ADR‐treated animals, our knowledge on the molecular underpinnings of this longevity phenotype remains limited. The first gene that was discovered to support ADR longevity is *cbp‐1*, encoding a histone acetyltransferase CREB binding protein, and its activity is required only in GABAergic neurons to drive ADR longevity (Cai et al. 2019; Zhang et al. 2009). Together with the observed upregulation of neuropeptide genes in axenically grown worms (Cai et al. 2022), this leads to the expectation that neuroendocrine signaling may play a crucial role in the ADR longevity phenotype.

The ability of an organism to maintain homeostasis in response to environmental challenges is fundamental for survival. In *C. elegans* , a neuroendocrine signaling network has evolved to relay signals within and among different tissues, facilitating the perception of food availability. Genetic studies highlight the significant role of sensory perception in homeostasis and lifespan regulation in *C. elegans* (Apfeld and Kenyon 1999; Bishop and Guarente 2007; Lee and Kenyon 2009) and *Drosophila melanogaster* (Libert et al. 2007). Sensory neurons, including gustatory, olfactory, thermosensory, and mechanosensory neurons, provide positive or negative inputs influencing lifespan, responding to various environmental cues (Alcedo and Kenyon 2004; Apfeld and Kenyon 1999; Lee and Kenyon 2009; Xiao et al. 2013). These different neurons can perceive a wide variety of environmental cues and modulate the activities of different peptides or steroid hormones, which in turn presumably affect homeostasis (Alcedo and Kenyon 2004; Antebi 2013; Kenyon et al. 1993; Xiao et al. 2013). Accumulating studies have revealed essential roles of both neurotransmitters and neuropeptides in remodeling sensory perception and food response in certain neural circuits (Artan et al. 2016; Leinwand and Chalasani 2013; Lin et al. 2017; Park et al. 2021). In *C. elegans* , seven types of neurotransmitters have been identified: acetylcholine (Ach), γ‐aminobutyric acid (GABA), dopamine, octopamine, serotonin (5‐HT), glutamate, and tyramine, which are packed into synaptic vesicles and released by exocytosis (Scalettar 2006; Weimer and Jorgensen 2003). Additionally, the *C. elegans* genome encodes a diverse array of bioactive peptides (neuropeptides), categorized into three families based on their conserved motifs: the insulin‐like peptides (ILPs) (Li et al. 2003; Pierce et al. 2001), the FMRFamide (Phe‐Met‐Arg‐Phe‐NH2)‐like peptides (FLPs) (Li 2005; Li et al. 1999), and neuropeptide‐like proteins (NLPs). Despite the complexity and abundance of neuropeptides, only four major proprotein convertase genes are present in *C. elegans* : *kpc‐1*, *egl‐3/kpc‐2*, *aex‐5/kpc‐3*, and *bli‐4/kpc‐4*. They are responsible for proteolysis and maturation of neuropeptides (Thacker et al. 1995; Thacker and Rose 2000), of which the release is part of the processing of distinct sets of sensory information into physiological responses that may optimize survival (Alcedo et al. 2013). These small molecules and peptides could coordinate different physiological activities such as egg laying, pharyngeal pumping, locomotion, and learning to maintain global homeostasis and maximize lifespan.

Here, we initiate a pilot screen of candidate genes involved in neurotransmitter and neuropeptide biology to reveal their potential involvement in ADR longevity. We found that BLI‐4 is specifically required for ADR longevity. BLI‐4, a member of the kex2/subtilisin‐like family of endoproteases, mainly cleaves collagen precursors (Thacker et al. 1995). Therefore, *bli‐4* mutants exhibit distinctive phenotypic features, including blistered adult cuticles and abnormalities in the molting cycle. Furthermore, we investigated the tissue‐specific role of BLI‐4 and demonstrated that BLI‐4 likely acts in neurons to modulate ADR longevity. The transcriptional activity of BLI‐4 in neurons prompted us to consider its potential association with the function of CBP‐1 in GABA neurons, a factor previously identified as essential for ADR longevity (Cai et al. 2019). To gain deeper insights into the molecular mechanisms governing ADR‐induced longevity, we conducted a proteomic analysis to identify downstream genes within this unidentified longevity program. This comprehensive approach allowed us to uncover potential effectors that contribute to the doubling of lifespan in response to ADR.

## 2 Methods

### 2.1 *C. elegans* and RNAi Strains

A list of *C. elegans* strains used in this study can be found in Table S1. *E. coli* strains (HT115) expressing dsRNA against target genes were obtained from the Vidal and Ahringer feeding libraries. The strain carrying the empty vector L4440 was used as a control. Strains were maintained at 20°C on nutrient agar (NA) plates seeded with *E. coli* OP50 as a food source.

### 2.2 PFA‐Killed Bacterial Food

A metabolically inactive food source, avoiding contamination of axenic medium, was obtained by treating *E. coli* bacteria with 0.5% paraformaldehyde (PFA) (Beydoun et al. 2021). Briefly, after overnight culturing *E. coli* , 32% PFA was added to the culture flasks to a 0.5% final concentration. PFA‐treated bacteria were shaken at 37°C for 1 h at 150 rpm, transferred to 50 mL conical tubes, and centrifuged at 3000 × *g* for 20 min. Supernatant was removed and washed with LB five times to remove residual PFA. After washing, the PFA‐treated bacteria were concentrated five times before seeding onto NGM (nematode growth medium) plates.

### 2.3 RNAi Treatment

To knock down genes of interest, RNAi by feeding was performed (Timmons et al. 2001). Briefly, synchronized worms were grown on NGM plates seeded with PFA‐killed *E. coli* OP50 to avoid contamination. Overnight bacterial cultures in LB supplemented with carbenicillin (50 μg/mL) at 37°C were seeded onto NGM plates containing IPTG (1 mM) and carbenicillin (25 μg/mL) and incubated at room temperature in the dark for 2 days to induce double‐stranded RNA. L4 larvae were placed on RNAi plates and incubated at 20°C for 5 days, after which worms were switched to their final dietary regimens.

### 2.4 Sample Preparation for Mass Spectrometry

Worms were subjected to RNAi treatment from L4 for 3 days and subsequently transferred to axenic medium for an additional 2 days of culturing. Approximately 1000 worms were harvested by rinsing with S‐buffer (salt‐buffer, 0.1 M NaCl + 1 M KH_2_PO_4_ + 1 M K_2_HPO_4_) and washed three times. *C. elegans* were pelleted by centrifugation, and all supernatant was removed. Worm pellets were flash frozen in liquid nitrogen and stored at −80°C. The pellet of worms was fast‐thawed and lysed by adding 400 μL RIPA lysis buffer, 5 μL complete antiprotease EDTA‐free solution, and 1 small scoop of 1.4 mm stainless steel beads to each sample. The samples were homogenized using a bead homogenizer, after which the samples were gently agitated on a rocker at 4°C for 30 min, followed by centrifugation at 16000 × *g* for 15 min to remove nuclei and cell debris. The protein concentration of the lysates was determined using BCA assays. Ten μg protein samples diluted with 50 mM triethylammonium bicarbonate (TEAB) were added to a final concentration of 2% sodium dodecyl sulfate (SDS). The samples were then reduced with 10 mM tris(2‐carboxyethyl) phosphine (TECP) and alkylated with 19 mM iodoacetamide (IAA). To this mixture, 2.5% phosphoric acid and 800 μL of binding/washing buffer were added. Protein digestion with trypsin was performed using the S‐Trap micro spin column digestion protocol (Thanou et al. 2023). In brief, 1 μg of trypsin was mixed with 10 μg of protein, adjusting the final volume to 100 μL using the TEAB/SDS/TECP/IAA buffer, followed by incubation at 37°C for 10 h. Subsequently, 50 mM TEAB was added, and the peptides were eluted twice, first by spinning the mixture and then with 40 μL 0.2% formic acid (FA) and 40 μL 50% acetonitrile (ACN). All elutes were combined and dried using a speedvac for subsequent mass spectrometry analysis.

### 2.5 Mass Spectrometry Analysis

Each sample was loaded on an HPLC fitted with a C18 trap column system coupled online with a timsTOF Pro operating in positive ion mode, coupled with a CaptiveSpray ion source (both from Bruker Daltonik GmbH, Bremen). The timsTOF Pro was calibrated according to the manufacturer's guidelines. The temperature of the ion transfer capillary was 180°C. The Parallel Accumulation–Serial Fragmentation data‐dependent acquisition (DDA) method was used to select precursor ions for fragmentation with 1 TIMS‐MS scan and 10 PASEF MS/MS scans, as described by Meier et al. (Meier et al. 2018). The TIMS‐MS survey scan was acquired between 0.70–1.45 V s/cm^2^ and 100–1700 m/z with a ramp time of 100 ms. The 10 PASEF scans contained on average 12 MS/MS scans per PASEF scan with a collision energy of 10 eV. Precursors with 1–5 charges were selected with the target value set to 20,000 a.u. and intensity threshold set to 2500 a.u. Precursors were dynamically excluded for 0.4 s. The timsTOF Pro was controlled by the TimsControl 4.1 software (Bruker Daltonik GmbH). The *.RAW files were exported and processed in PEAKS Online X. The files were searched using target‐decoy matching with the *C. elegans* Uniprot database, with the false discovery rate set at 1%. Trypsin was indicated as the enzyme, and up to 2 miscleavages were allowed. Carbamidomethylation, deamidation, and oxidation were set as a fixed modifications. Label‐Free Quantification (LFQ) and Match Between Runs were used using default settings.

### 2.6 Functional Analysis and Data Visualization

The raw data generated by the mass spectrometer were analyzed using quantitative proteomics software FragPipe, including the MSFragger search engine (Yu et al. 2023). The protein sequence of *C. elegans* was sourced from Uniprot for reference. Protein quantification was performed using the label‐free quantification (LFQ) algorithm. The resulting LFQ intensity was log_2_‐transformed and imputed for missing values in Perseus software (Tyanova et al. 2016). Significance of enrichment was measured by a two‐sample Student's *t* test. Gene ontology (GO) analysis was conducted through the online bioinformatic tool, the Database for Annotation, Visualization, and Integrated Discovery (DAVID) (Huang da et al. 2009). GraphPad Prism 9 was used for data visualization.

### 2.7 Lifespan Assay

Lifespan assays were conducted as described previously (Castelein et al. 2014). In brief, worms were cultured on standard NGM plates, supplemented with PFA‐killed *E. coli* OP50 bacteria, until reaching the L4 larval stage. RNAi was performed on L4 worms for 5 days prior to their transfer to experimental conditions. One hundred worms were placed into small screw‐cap tubes (3–5 worms per tube) containing 0.3 mL of axenic medium (3% yeast extract +3% soy peptone +0.05% hemoglobin +5 μg/mL cholesterol), and around one hundred worms were placed on small NGM plates (10 per plate) seeded with *E. coli* OP50 as fully fed (FF) control. For the lifespan assay of worms in axenic dilution, the standard axenic medium (defined as 100% axenic) was diluted four‐fold to create a 25% axenic medium using salt‐buffer (S‐buffer: 0.1 M NaCl +1 M KH2PO4 + 1 M K2HPO4), which is regarded as 0% axenic. The methods for bacterial dilution in liquid (bDR) (Bishop and Guarente 2007) and on solid plates (sDR) (Greer et al. 2007), as well as bacterial deprivation on solid plates (DD) (Kaeberlein et al. 2006), were performed as previously described. Briefly, *E. coli* OP50 bacteria were diluted 1000‐fold or deprived and resuspended in S‐buffer to inhibit growth. L4 stage worms were then transferred to the appropriate medium. To prevent progeny production, a final concentration of 100 μM FUdR was added. Survival was scored every other day. Worms on NGM plates were considered dead if they did not respond to gentle prodding with a platinum wire. In liquid conditions, worms were scored dead if no movement was detectable, even after the tubes were gently tapped. Worms that died of protruding vulva or crawling off the plates were censored. All lifespan experiments were carried out at a temperature of 20°C.

Lifespan data was analyzed with the online application for survival analysis (OASIS) as described by (Yang et al. 2011). Presentation of lifespan data was accompanied by mean ± s.e.m., and *p*‐values were calculated using the log‐rank (Mantel–Cox) method. Graphs were made using GraphPad Prism 9. COX proportional hazards analysis was used to evaluate whether the interaction of terms (medium × genotype) significantly impacts survival. Lifespan data and statistics for all experiments are provided in Tables S3–S5.

## 3 Results

### 3.1 Neurotransmitter Signaling Has a Limited Role in ADR‐Induced Longevity

In our previous studies, we demonstrated that ADR‐mediated longevity depends on neuronal CBP‐1 (Cai et al. 2019) and we observed upregulation of numerous neuropeptides in axenically cultured worms (Cai et al. 2022), suggesting a potential role for neuronal signaling in the ADR longevity process. We first aimed to elucidate the role of neurotransmission in the regulation of ADR lifespan by conducting lifespan assays on *unc‐13* deficient worms, in which neurotransmitter vesicle release is compromised. Due to the presence of a severe uncoordinated phenotype observed in axenically cultured *unc‐13* mutants, neuronal RNAi knockdown was used to investigate the effect of *unc‐13* on ADR lifespan regulation. Inhibition of *unc‐13* showed no notable change in the lifespan of FF or ADR (Figure S1a). This finding contrasts with prior research indicating that *unc‐13* deficiency leads to increased lifespan when fed normal bacterial food (Munoz and Riddle 2003). Nonetheless, we cannot exclude the role of neurotransmission entirely in ADR longevity because, despite using a neuron‐specific RNAi strain, knockdown efficiency may have been insufficient. To further investigate the potential contribution of neurotransmitters to the extended lifespan observed in axenically cultured worms, we examined mutant animals harboring defects in major neurotransmitter pathways, including *cha‐1* (acetylcholine), *tph‐1* (serotonin), *cat‐2* (dopamine), *tdc‐1* (tyramine), *tbh‐1* (octopamine), *eat‐4* (glutamate), and *unc‐25* (GABA) (Alfonso et al. 1994; Alkema et al. 2005; Jin et al. 1999; Lee et al. 1999; Lints and Emmons 1999; Sze et al. 2000). None of these mutants significantly reduced the ADR longevity effect (Figure S1b–h, Tables S3 and S4). Notably, *tbh‐1(n3247)* mutation, leading to a defect in the synthesis of octopamine, leads to synergistic lifespan extension under ADR. Furthermore, inhibition of *cat‐1*, a gene essential for the transport of biogenic amine neurotransmitters, also has a negligible impact on ADR‐mediated lifespan extension (Table S3). Overall, the results indicate that, while ADR exerts its effects on lifespan extension via neurons, the involvement of neurotransmitters in this process is not crucial.

### 3.2 Inhibition of BLI‐4 Abolishes ADR‐Mediated Lifespan Extension

**FIGURE 1:**
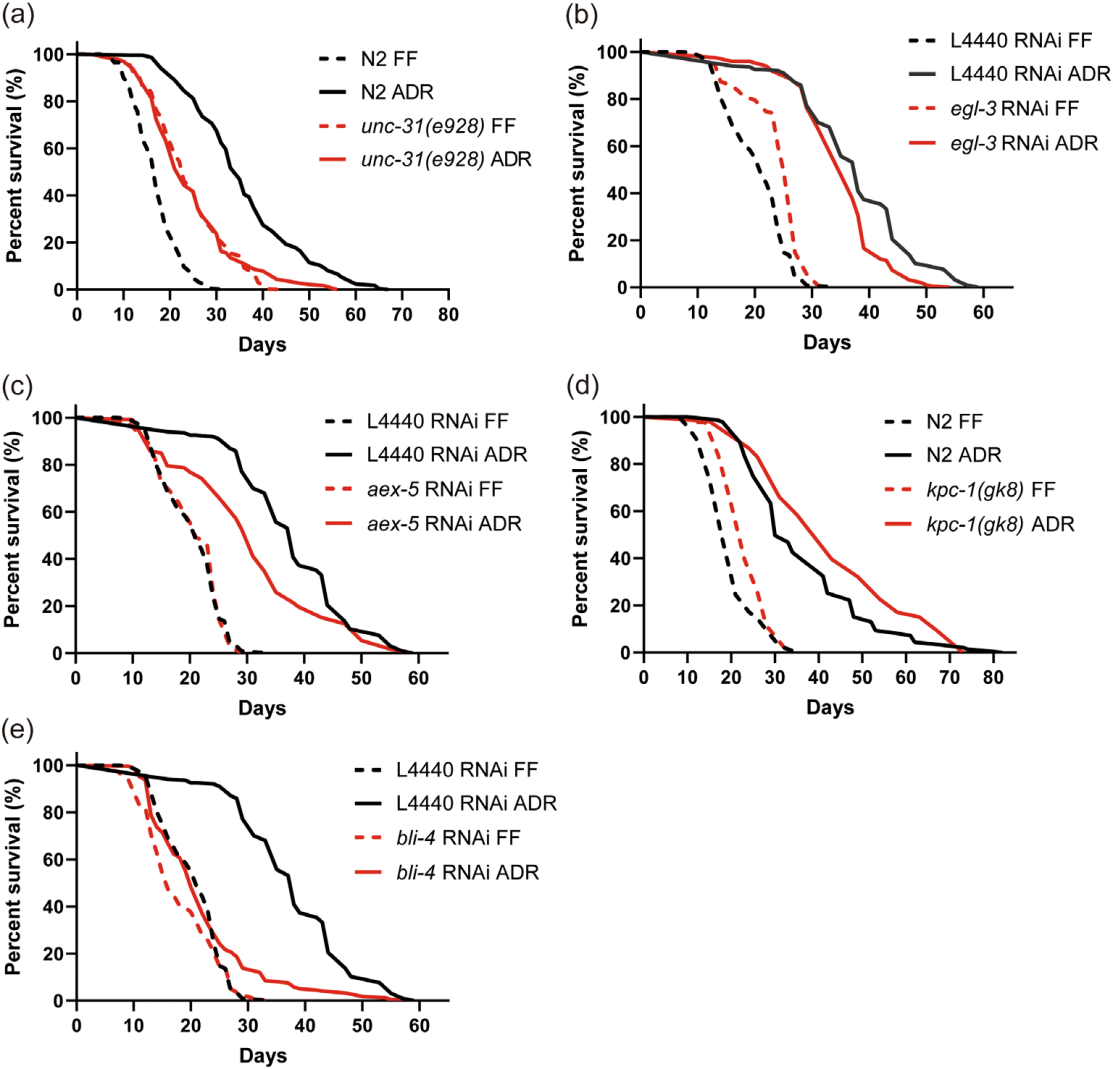
RNAi inhibition of *bli‐4* abolishes ADR‐mediated lifespan extension. Survival curves of worms deficient in (a) the neuropeptide secretion gene *unc‐31(e928)*, and four major proprotein convertase genes (b) *egl‐3* RNAi, (c) *aex‐5* RNAi, (d) *kpc‐1(gk8)*, (e) *bli‐4* RNAi under FF and ADR conditions. ADR = axenic dietary restriction, FF = fully fed. *p‐*values were calculated by log‐rank tests with Bonferroni correction. See survival statistics in Tables S3 and S4.

Apart from neurotransmitters, neuropeptides are another large group of neuronal signaling molecules. To investigate whether these are required for ADR‐induced longevity, we conducted a small genetic screen targeting a list of neuropeptide genes known to be upregulated under ADR (Cai et al. 2022). However, this lifespan screen did not reveal any neuropeptides that significantly affect ADR‐induced longevity (Table S5). Neuropeptides are typically derived from larger precursor molecules, which are cleaved by specific proprotein convertase enzymes to generate mature neuropeptides. The *C. elegans* genome is known to contain a minimum of 113 neuropeptide genes, which include 40 INS‐, 31 FLP‐, and 42 NLP‐related genes, encoding approximately 250 distinct neuropeptides (Li and Kim 2008). These neuropeptides are processed by four proprotein convertases: *kpc‐1, egl‐3/kpc‐2, aex‐5/kpc‐3*, and *bli‐4/kpc‐4* (Thacker and Rose 2000). Therefore, we employed an alternative approach by individually knocking down or knocking out each of these convertases, as well as *unc‐31*, an activator protein for neuropeptide secretion (Speese et al. 2007). Consistent with a previous study (Ailion et al. 1999), *unc‐31(e928)* resulted in lifespan extension in monoxenic conditions, while this mutant showed a significantly reduced ADR longevity phenotype (Figure 1a). This result highlights that neuropeptides are involved in regulating ADR‐induced longevity. We next tested the four main proprotein convertases. Only RNAi knockdown of *bli‐4* completely abolished ADR‐induced lifespan extension (Figure 1b–e, Table S3). Although *bli‐4* seems to be involved in lifespan extension under ADR, it is not significantly upregulated under these conditions (Figure S2). To further validate these RNAi results, we conducted a survival assay using viable mutants of proprotein convertases. None of these mutations resulted in a significant reduction of longevity induced by ADR (Figure S3). Additionally, in line with previously published data, we observed an extended lifespan in *egl‐3(ok979)* mutants under FF conditions and ADR conditions (Hamilton et al. 2005). As axenically cultured *bli‐4(e937)* mutants did not exhibit a shorter lifespan compared to N2, we hypothesized that *bli‐4* isoforms that are unaffected by the *e937* deletion allele are involved in ADR longevity.

### 3.3 BLI‐4 Is Essential in the Neurons for ADR Mediated Longevity

**FIGURE 2:**
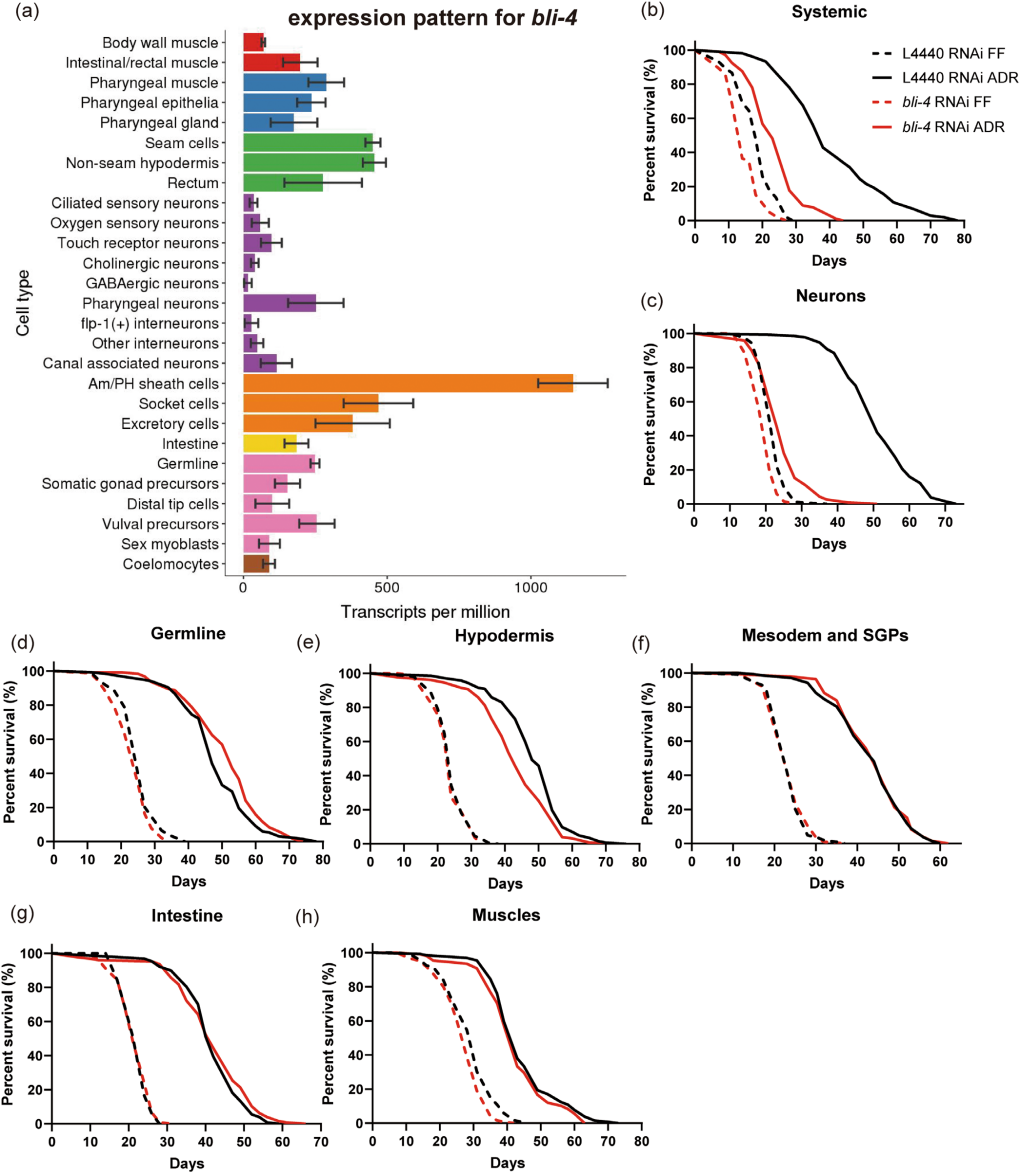
*bli‐4* is required in neurons to support ADR‐induced lifespan extension. (a) expression pattern of *bli‐4* in different cell types (GExplore). Tissue specific knockdown of *bli‐4* in (b) systemic, (c) neurons, (d) germline, (e) hypodermis, (f) mesoderm and somatic gonadal precursors (SGPs), (g) intestine, (h) muscles. ADR = axenic dietary restriction, FF = fully fed. *p‐*values were calculated by log‐rank tests with Bonferroni correction. See survival statistics in Tables S3 and S4.

Single cell RNA sequencing (scRNAseq) and a *bli‐4* transcriptional reporter revealed that *bli‐4* is mainly expressed in the epidermis, pharynx, intestine, germline, and glia (Figure 2a) (Birnbaum et al. 2023; Cao et al. 2017; McKay et al. 2003). As we wondered what types of tissues are required for BLI‐4 to promote lifespan extension under ADR conditions, we conducted survival assays targeting *bli‐4* knockdown tissue‐specifically during the initial 5 days of adulthood. The tissues included the hypodermis, germline, intestine, mesoderm, and somatic gonadal precursor cells, neurons, and glia. Given that CBP‐1 has been shown to be crucial in GABAergic neurons specifically to extend lifespan under ADR (Cai et al. 2019), we hypothesize that BLI‐4 might also play a significant role in neurons to regulate ADR longevity. Indeed, a pan‐neuronal knockdown of *bli‐4* in two independent strains largely abolished the lifespan extension observed under ADR conditions (Figure 2c, Figure S4a). This outcome closely mirrored the lifespan effect seen with systemic knockdown of *bli‐4* (Figure 2b). Conversely, knockdown of *bli‐4* in other tissues exhibited minimal or no impact on lifespan (Figure 2d–h, Table S3). This suggests that BLI‐4 mainly functions in neurons to regulate ADR longevity.

The *C. elegans* hermaphrodite contains 302 neurons, classified into seven neurotransmitter‐based subclasses (acetylcholine, serotonin, dopamine, tyramine, octopamine, glutamate and GABA) (Chase and Koelle 2007; Hobert 2005). We next aimed to determine whether BLI‐4, like CBP‐1, specifically functions in GABA neurons or any other neuron type to mediate ADR longevity. We knocked down *bli‐4* in the cholinergic, glutamatergic, dopaminergic, and GABAergic neurons (Firnhaber and Hammarlund 2013). Against our expectation, *bli‐4* does not mediate ADR longevity in any of these neuron types (Figure S4b–e). BLI‐4 is highly expressed in Am/PH sheath cells (Cao et al. 2017; Hutter and Suh 2016), but our results suggest that BLI‐4's presence in these cells is also not essential for ADR longevity (Figure S4f). It appears that BLI‐4 may exert its lifespan‐prolonging effects in other, yet‐to‐be‐identified neuron types. Alternatively, given the known leakiness of neuron‐specific RNAi strains (Gahlot and Singh 2024), BLI‐4 may act in non‐neuronal tissue other than our tested glia, germline, hypodermis, intestine, muscle or mesoderm and somatic gonad precursors.

### 3.4 BLI‐4 Is Specific to ADR Mediated Longevity

**FIGURE 3:**
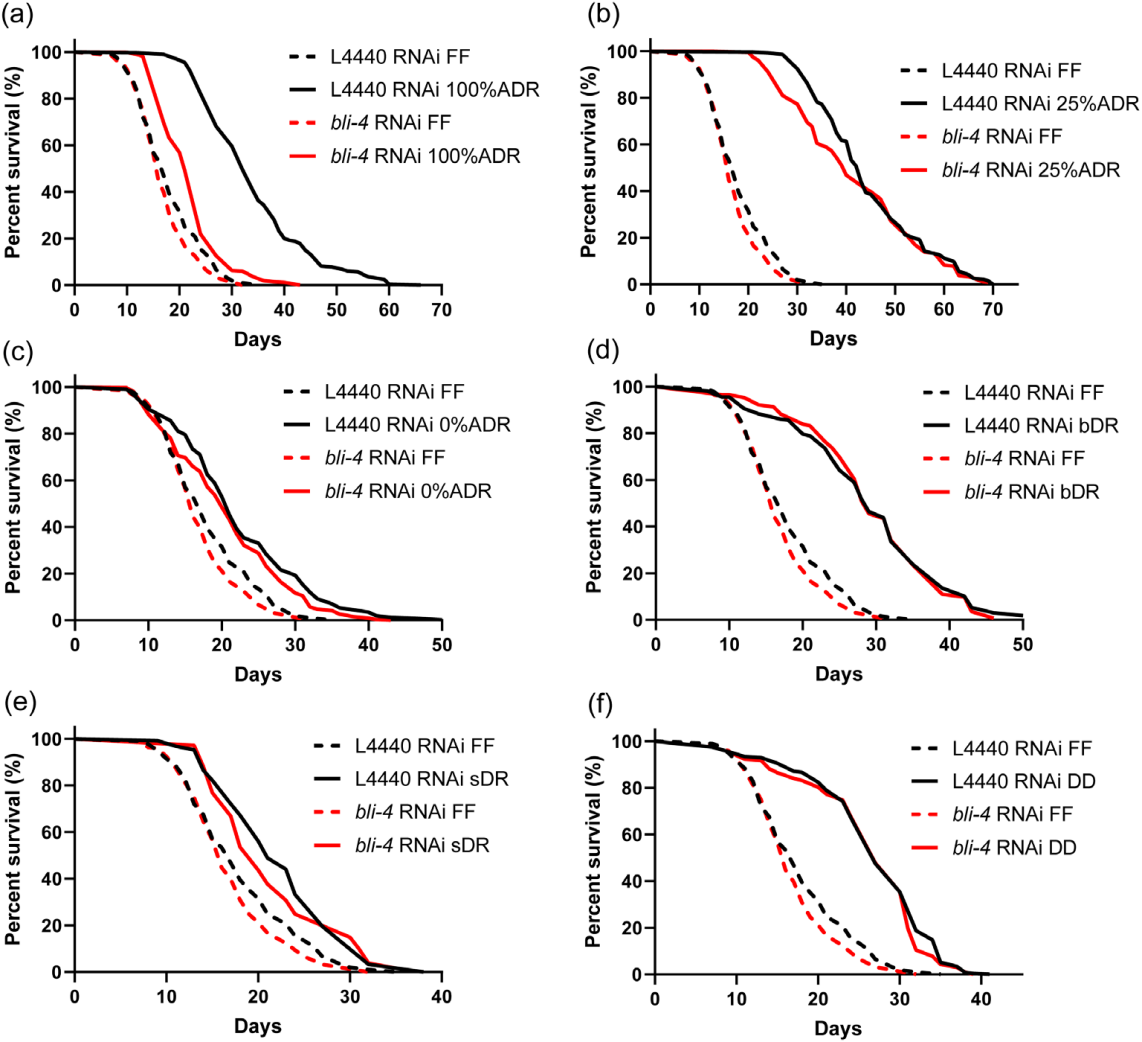
Effect of *bli‐4* knockdown on lifespan in different dietary regimens. Survival of worms in (a) 100% ADR, (b) 25% ADR, (c) 0% ADR, (d) bDR, bacterial dilution in liquid culture, (e) sDR, bacterial dilution on agar plates, (f) DD, dietary deprivation on solid plates. Salt buffer (S‐buffer, 0.1 M NaCl +1 M KH_2_PO_4_ + 1 M K_2_HPO_4_) was used to dilute the standard 100% ADR to the specified concentrations. 1000‐fold bacterial dilution food was used as bDR and sDR. ADR = axenic dietary restriction, FF = fully fed. *p‐*values were calculated by log‐rank tests with Bonferroni correction. See survival statistics in Tables S3 and S4.

As CBP‐1 is implicated in food sensing and regulation of ADR‐induced longevity, we hypothesized that axenic or bacterial signals may contribute to this phenotype (Cai et al. 2019). To test whether BLI‐4 regulates these signals, we exposed animals to different DR regimens, including axenic medium dilution, bacterial dilution, and food deprivation. Wild‐type animals consistently exhibited an extended lifespan over all DR regimens, ranging from 24% to 148% (Figure 3, Table S3). In contrast, *bli‐4* RNAi knockdown specifically impeded longevity induced by standard axenic medium, suggesting the importance of a relatively strong food cue for the activation of the BLI‐4‐mediated longevity program (Figure 3). As longevity caused by different diets depends on a variety of partially overlapping gene sets (Greer and Brunet 2009), we further analyzed the contribution of some DR‐related genes to longevity in ADR dilutions. We found that the energy sensors AMPK/*aak‐2* and *sir‐2.1*, and the transcriptional regulator *cbp‐1* support longevity across the entire axenic dilution series. The stress‐related transcription factors *daf‐16* and *hlh‐30* only support longevity under severe nutrient scarcity (0% and 25% axenic medium). *bli‐4* only supported the lifespan doubling effect of standard axenic medium (Figure S5). The highly diet‐specific role of BLI‐4 in longevity confirms our suspicion that lifespan extension induced by ADR is quite unique from that of other DR methods (Castelein et al. 2014). These results underscore the significance of the exact nutritional status as a crucial determinant in the precise molecular mechanisms governing ADR‐mediated lifespan extension.

### 3.5 Multiple BLI‐4 Isoforms Contribute to ADR Longevity

**FIGURE 4:**
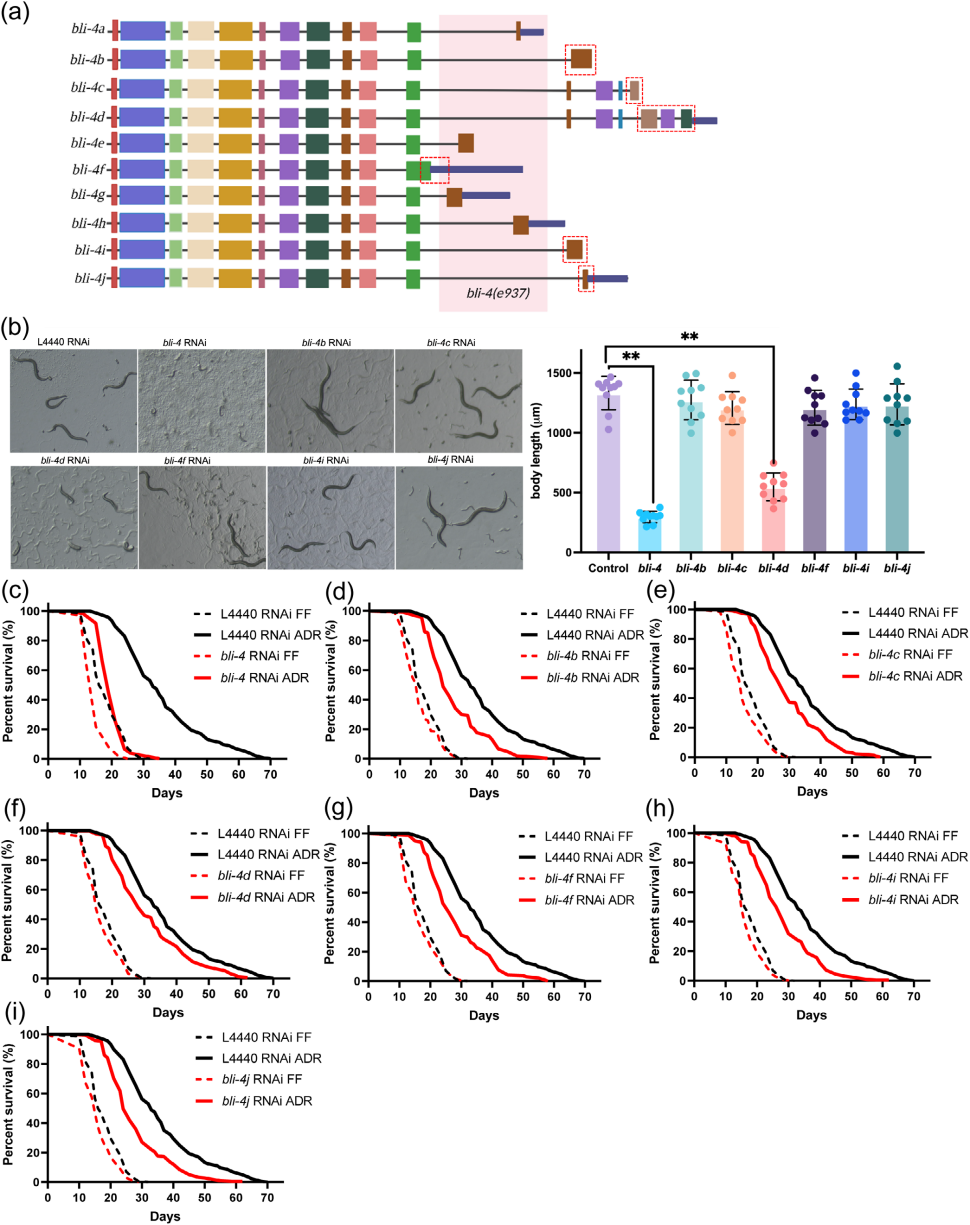
*bli‐4* isoforms have overlapping effects on ADR‐mediated longevity. (a) alternative splicing of *bli‐4* results in 10 isoforms. the deletion allele *bli‐4(e397)*, targeting isoforms *a, e, g* and *h*, is indicated in pink; RNAi target sites and exon specific mutations are indicated with a red dashed border. (b) developmental arrest in worms treated with *bli‐4d* RNAi. Body lengths of isoform knockdowns were compared with controls using two‐tailed *t*‐tests (*n* = 10). *p < 0.05; **p < 0.01; ***p < 0.001. (c–i) Survival curves of worms submitted to *bli‐4* RNAi (c), and RNAi of *bli‐4* isoform *b* (d), isoform *c* (e), isoform *d* (f), isoform *f* (g), isoform *i* (h), and isoform *j* (i). ADR = axenic dietary restriction, FF = fully fed. *p‐*values were calculated by log‐rank tests with Bonferroni correction. See survival statistics in Tables S3 and S4.

The proprotein convertase BLI‐4 is a serine endoprotease and its primary function is to cleave pro‐collagen and neuropeptides into their mature form (Thacker et al. 2000). *bli‐4* has up to 10 different isoforms which share 11 exons containing the catalytic domain (Figure 4a). *bli‐4*(*e937*) mutants have a blistered adult cuticle, compatible with the function of BLI‐4 as an enzyme cleaving cuticle collagens (Thacker et al. 1995). While *bli‐4* RNAi knockdown abolishes ADR‐induced longevity, *bli‐4(e397)* mutation does not (Figure S3b). *e397* is a deletion removing exons unique to *bli‐4* isoforms *a, e, g, and h* (Peters et al. 1991). Therefore, these isoforms can be excluded from playing a role in ADR‐mediated longevity. To gain further insights into the longevity contribution of the other six *bli‐4* isoforms, we used exon‐specific RNAi and isoform‐specific exon deletion by CRISPR‐Cas9 (Figure 4a, Table S2).

General RNAi knockdown of *bli‐4* (all isoforms) from L1 larvae leads to developmental arrest at the L1/L2 stage. Interestingly, knockdown of only the *bli‐4d* isoform is sufficient to obtain the same phenotype, while no arrest was observed upon knockdown of all other isoforms (*b*, *c*, *f*, *i*, and *j*) (Figure 4b). Only *bli‐4d* contains a cysteine‐rich domain similar to that found in mammalian furin, suggesting this domain is particularly important for processing substrates during early larval stages (Birnbaum et al. 2023). To investigate the involvement of *bli‐4* isoforms in ADR longevity, an exon‐specific RNAi approach was employed from the L4 stage for a duration of 5 days, after which lifespan was analyzed in axenic medium. Except for *bli‐4c* and *bli‐4d*, all other tested *bli‐4* isoforms (*b, f, i*, and *j*) partially influence the ADR longevity (Figure 4d–i, Table S3, S6). This indicates that the control of ADR‐mediated longevity and development by *bli‐4* are distinct processes. The mean lifespan of *bli‐4* RNAi worms under ADR conditions was reduced by 44.16%. The specific reductions in longevity for each *bli‐4* isoform RNAi under ADR were 23.19% (*bli‐4b*), 19.03% (*bli‐4f*), 20.69% (*bli‐4i*), and 22.66% (*bli‐4j*) respectively (Table S3). The contribution of each isoform to ADR longevity was largely corroborated by lifespan analyses of isoform mutants generated by CRISPR‐Cas9 (Figure S6; Tables S4 and S6). Only one inconsistency appeared: mutation of *bli‐4c* led to a significant decrease in ADR longevity, while this was not the case for *bli‐4c* RNAi. Hence, our results for *bli‐4c* remain inconclusive. Overall, these findings suggest that several *bli‐4* isoforms have overlapping functions in modulating longevity under ADR conditions.

### 3.6 GOLG‐2 Is Required for BLI‐4 Regulated ADR Longevity

**FIGURE 5:**
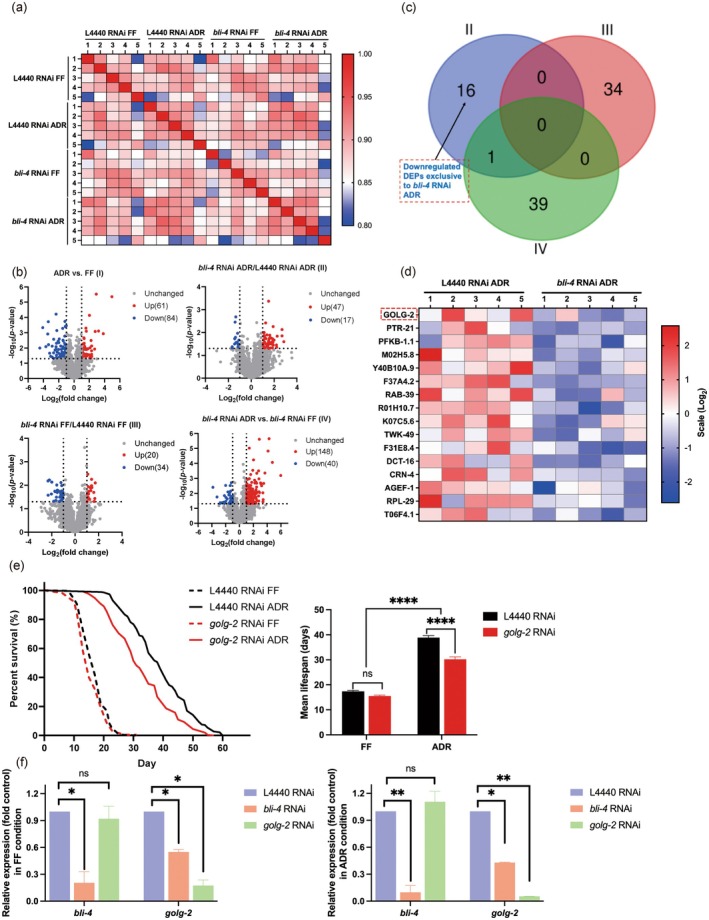
GOLG‐2 is likely a downstream protein involved in *bli‐4*‐mediated ADR longevity. (a) matrix representation of Pearson correlation values of the protein abundance across five biological replicates. (b) Volcano plots of differentially expressed proteins (DEPs) in following four comparisons: L4440 RNAi ADR vs. L4440 RNAi FF (I), *bli‐4* RNAi ADR vs. L4440 RNAi ADR (II), *bli‐4* RNAi FF vs. L4440 RNAi FF (III), and *bli‐4* RNAi ADR vs. *bli‐4* RNAi FF (IV) data sets. (c) Venn diagram showing the number of identified downregulated DEPs in *bli‐4* RNAi worms. (d) Heatmap for 16 proteins specifically downregulated by *bli‐4* RNAi in ADR conditions. (e) Effect of *golg‐2* knockdown on worm survival in ADR and FF conditions. ADR = axenic dietary restriction, FF = fully fed. *p‐*values were calculated by log‐rank tests with Bonferroni correction. See survival statistics in Tables S3 and S4. (f) Relative expression levels of *bli‐4* and *golg‐2* under different RNAi conditions in FF (left panel) and ADR (right panel) states. Error bars represent mean ± SEM; statistical analysis by one‐way ANOVA (*n* = 2) (*p < 0.05, **p < 0.01, ns, not significant).

To find downstream actors of BLI‐4 in ADR‐induced longevity we conducted a liquid chromatography‐MS (LC–MS) label‐free quantitative proteomics analysis of wild type (L4440 RNAi) and *bli‐4* RNAi animals in FF and ADR conditions respectively. As we wanted to focus on the physiological effects of *bli‐4* knockdown and the ADR diet in young adult worms, we first aimed to minimize the duration of the RNAi treatment (carried out under FF conditions). We tested RNAi treatments for a period of 2 to 5 days (starting from L4) and subsequently assessed knockdown efficiency and effect on lifespan (Figure S7). A 3‐day RNAi exposure period appeared optimal to obtain efficient *bli‐4* knockdown, a significant effect on lifespan, and worms that were still young after 2 days of ADR treatment. Five replicates were run for proteomic analysis, and these showed high reproducibility as indicated by Pearson correlation analysis (*r* > 0.93) over diets (ADR vs. FF) and genes (L4440 vs. *bli‐4*) (Figure 5a). We created four comparison groups: L4440 RNAi ADR vs. L4440 RNAi FF (I), *bli‐4* RNAi ADR vs. L4440 RNAi ADR (II), *bli‐4* RNAi FF vs. L4440 RNAi FF (III), and *bli‐4* RNAi ADR vs. *bli‐4* RNAi FF (IV). Bioinformatic analysis revealed that with the cutoff at ≥ 2 or ≤ 0.5 fold change and *p* < 0.05, there are 145 (61 upregulated and 84 downregulated), 64 (47 upregulated and 17 downregulated), 54 (20 upregulated and 34 downregulated), and 188 (148 upregulated and 40 downregulated) differentially expressed proteins (DEPs) in the I, II, III, and IV comparison groups, respectively (Figure 5b). These results indicate that ADR can induce relatively strong proteome changes compared to *bli‐4* alone, and animals treated with *bli‐4* RNAi exhibited only modest proteomic changes relative to the empty‐vector control in axenic culture.

We found 16 DEPs may be key players in *bli‐4*‐associated ADR longevity (Figure 5c,d). Only knockdown of *golg‐2* was found to partially, yet significantly, decrease the longevity effect of ADR (Figure 5e). Additionally, the expression level of *golg‐2* was significantly reduced under *bli‐4* RNAi conditions, whereas *bli‐4* expression remained unchanged upon *golg‐2* knockdown (Figure 5f). These findings suggest that *golg‐2* likely acts downstream of *bli‐4* in the ADR‐induced longevity pathway. Given the known role of GOLG‐2 in protein and lipid trafficking (Jung et al. 2021), it can be inferred that proper Golgi function, potentially regulated by *bli‐4*, is essential for the lifespan extension observed in ADR.

Finally, knockdown of *bli‐4* may result in a compensatory upregulation of its target proteins via feedback loops. *bli‐4* RNAi causes the upregulation of 47 proteins in ADR conditions, 23 (49%) of which contain at least one potential BLI‐4 cleavage site RXXR (Table S7) (Leinwand and Chalasani 2013), which is not an overrepresentation compared to the occurrence of this motif in the entire proteome (60%). The functions of these 23 proteins are diverse but include three proteins involved in Ca^2+^ homeostasis: the calmodulin‐binding F38H12.3 and AKT‐1, and the endoplasmic reticulum Ca^2+^‐transporter SCA‐1. Upregulation of SCA‐1 is required for *eat‐2* and *ifg‐1* mediated longevity (Rogers et al. 2011). Also, two collagens are upregulated, which may be linked to the collagen remodeling properties of BLI‐4, the known link of collagens to longevity (Ewald et al. 2015) and the better maintenance of cuticle structure in worms that age under ADR conditions (Wu et al. 2024). However, we could not link the putative collagen processing isoform *bli‐4d* (Figure 4b) to ADR longevity (Figure 4f).

## 4 Discussion

The molecular mechanism of ADR‐mediated lifespan extension in *C. elegans* is likely unique as it is independent of most longevity mediators identified earlier, including transcription factors SKN‐1/Nrf2, PHA‐4/FOXA, DAF‐16/FOXO, HSF‐1/HSF, HLH‐30/TFEB, as well as their downstream processes such as mitochondrial function and autophagy (Cai et al. 2017, 2022; Castelein et al. 2014; Houthoofd et al. 2003). This study uncovers the role of BLI‐4 in ADR longevity. BLI‐4 is one of the four *C. elegans* furin/proprotein convertases that cleave secreted or transmembrane substrates to generate mature bioactive factors, particularly neuropeptides (Thacker and Rose 2000). It is the most complex proprotein convertase that produces at least 10 isoforms through alternative splicing. We found that the *d* isoform is involved in development while *b*, *f*, *i*, and *j* isoforms all partially support ADR longevity. This dual role of isoforms in development and longevity is reminiscent of that of DAF‐16 (Kwon et al. 2010; Lee and Lee 2022), which is known not to be involved in ADR longevity (Houthoofd et al. 2003).

Like CBP‐1 (Cai et al. 2019), BLI‐4 too likely exerts its effects within the neural circuitry to support ADR longevity. Neurons sense nutritional cues and act in signal transduction to adjust organismal physiology in response to DR. For example, the sensation of the bacterial metabolite diacetyl shortens lifespan in food‐deprived worms (Park et al. 2021). Also, the activation of the NRF2 homolog SKN‐1 in two sensory ASI neurons is required for lifespan extension under a regimen of bacterial dilution (Bishop and Guarente 2007; Tullet et al. 2008). However, SKN‐1 is not implicated in ADR longevity (Castelein et al. 2014). Hence, BLI‐4 emerges as a player in a novel neuropeptide pathway that robustly promotes longevity in *C. elegans* , but the neuropeptides involved remain to be identified. BLI‐4 has been recognized as a key processor of subsets of insulin‐like peptide (ILP) precursors (Hung et al. 2014; Leinwand and Chalasani 2013), but ILPs are unlikely candidates as their receptor DAF‐2 and downstream effector DAF‐16 are not relevant to ADR longevity (Houthoofd et al. 2003). In the gustatory ASEL neuron, BLI‐4 processes INS‐6 for chemosensation (Leinwand and Chalasani 2013). However, this peptide shortens lifespan by decreasing DAF‐16 activity rather than extending it (Artan et al. 2016). In agreement with this, we here show that *ins‐6* knockdown in ADR worms does not shorten lifespan. BLI‐4 is also implicated in the cleavage of specific FMRFamide‐like peptides (FLPs) and neuropeptide‐like proteins (NLPs), some of which expression is upregulated under ADR conditions (Cai et al. 2022), but none of these seemed to be individually involved in ADR longevity. Previous microarray analysis demonstrated the upregulation of NLP‐7 in response to DR in a chemically defined medium (CeMM) (Szewczyk et al. 2006), but also *nlp‐7* is not involved in ADR longevity (Castelein et al. 2014). This indicates that BLI‐4 may produce other types of neuropeptides to support ADR longevity or that some of the BLI‐4 dependent peptides are functionally redundant to extend lifespan under ADR conditions.

Perturbation of *bli‐4* in ADR worms downregulates GOLG‐2, a protein predicted to function in Golgi complex organization, and of which knockdown also leads to reduced ADR longevity. The Golgi complex is central to sorting, modification, and trafficking of lipids and proteins (Goldfischer 1982). Given that BLI‐4 likely cleaves neuropeptides in the secretory pathway, the function of GOLG‐2 may be directly linked to this process. However, currently it is still unclear whether both BLI‐4 and GOLG‐2 act in the same tissues to promote ADR longevity. Also, the effect of *golg‐2* knockdown on ADR longevity is weaker than that of *bli‐4* knockdown. This partial effect may be the result of the interaction of *golg‐2* with only a limited number of *bli‐4* isoforms (that each partially contribute to the ADR longevity phenotype). GOLG‐2 may also act in cells that are downstream of the neuropeptide signal that promotes ADR longevity.

Recently, it was found that glucose restriction extends lifespan in *C. elegans* via neuronal AAK‐2a activity resulting in a neuropeptide signal. This signal triggers peripheral activity of NHR‐49 that promotes lipid homeostasis and activates desaturases. The resulting increase in membrane fluidity may promote longevity (Jeong et al. 2023). The undefined axenic medium used in this study is based on a mixture of soy peptone and yeast extract and therefore is very rich in peptides and amino acids while containing only limited amounts of glucose (Lenaerts et al. 2008), thereby potentially mimicking a glucose‐restricted diet. We showed previously that ADR longevity partially depends on *aak‐2* (Castelein et al. 2014) and in this study we found that neuronal *bli‐4*‐dependent neuropeptide processing is key to ADR longevity. Hence, it is plausible that the AAK‐2a/neuropeptide/NHR‐49 pathway is activated during ADR. However, the role of NHR‐49 in ADR longevity is less unequivocal. In axenic medium, BLI‐4 dependent upregulation of NHR‐49 was found in the proteomics experiment but could not be confirmed by qPCR (Figure S8a). Also, ADR longevity was reduced significantly by *nhr‐49* RNAi, but not by *nhr‐49* mutation (Figure S8b,c). Nevertheless, the role of NHR‐49 in lifespan extension, especially in neurons, has been confirmed in various dietary and genetic models of DR, including glucose depletion, starvation, and *eat‐2* mutation (Chamoli et al. 2014; Jeong et al. 2023; Marcellino et al. 2018). Investigations into the nervous system reveal that NHR‐49 governs neuroendocrine gene expression in response to peripheral lipid signals (Savini et al. 2022). However, this neuroendocrine pathway involves NLP‐11, for which we here show that it has no effect on ADR longevity.

In conclusion, we show that ADR longevity likely depends on the generation of a neuropeptide signal by BLI‐4. This may be mediated by the specific nutrient composition of the axenic medium and likely linked to GOLG‐2, which localizes in the Golgi to help protein and lipid trafficking, partially supporting lifespan extension. The further characterization of the role of Golgi function in ADR longevity represents a valuable direction for future research. These insights not only highlight the pivotal role of BLI‐4 in the longevity landscape but also open avenues for targeted interventions that mimic the beneficial effects of ADR, potentially offering novel strategies for aging and metabolic health.

## Author Contributions

Conceptualization: Ping Wu, Lieselot Vandemeulebroucke, and Huaihan Cai. Methodology: Ping Wu, Lieselot Vandemeulebroucke, and Huaihan Cai. Data curation: Ping Wu, Lieselot Vandemeulebroucke, and Huaihan Cai. Formal analysis: Ping Wu. Writing – original draft: Ping Wu. Visualization: Ping Wu. Supervision: Bart P. Braeckman. Project administration: Bart P. Braeckman. Funding acquisition: Bart P. Braeckman. All authors have read and agreed to the published version of the manuscript.

## Conflicts of Interest

The authors declare no conflicts of interest.

## Supporting information


Figures S1–S8.

Tables S1–S7.

## Data Availability

The mass spectrometry proteomics data has been deposited to the ProteomeXchange Consortium via the PRIDE (Perez‐Riverol et al. 2022) partner repository with the dataset identifier PXD053074.
