# Supplementary material for: The Proprotein Convertase BLI‐4 Is Required for Axenic Dietary Restriction Mediated Longevity in Caenorhabditis elegans
Source: Aging Cell. 2025 Apr 8;24(7):e70058. doi: 10.1111/acel.70058 (PMC12266778; doi:10.1111/acel.70058)
Supplement: Supplementary file 1 — Figures S1–S8. [file ACEL-24-e70058-s001.docx]

**Supplementary figures**


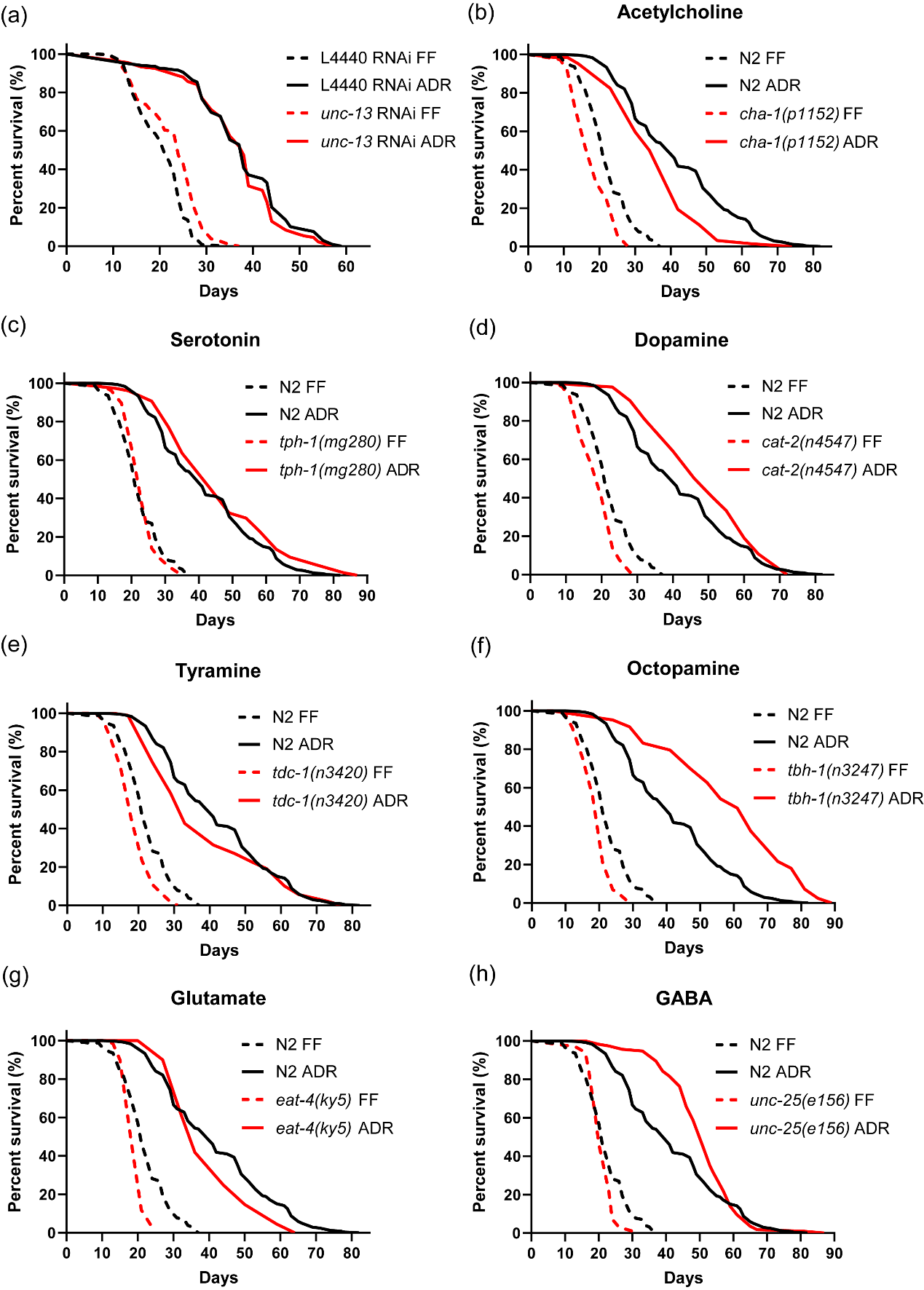


Figure S1. Neurotransmitter synthesis is not required for ADR-induced longevity. Survival of worms with defects in (a) *unc-13* RNAi: neurotransmitter release, Neuro-specific RNAi strain TU3401 was used. (b) *cha-1*: acetylcholine synthesis, (c) *tbh-1*: serotonin, (d) *cat-2*: dopamine, (e) *tdc-1*: tyramine, (f) *tbh-1*: octopamine, (g) *eat-4*: glutamate, and (h) *unc-25*: GABA under FF and ADR conditions. FF = fully fed, ADR = axenic dieatry restriction. *P-*values were calculated by log-rank tests with Bonferroni correction. See survival statistics in Supplementary Table 3&4.


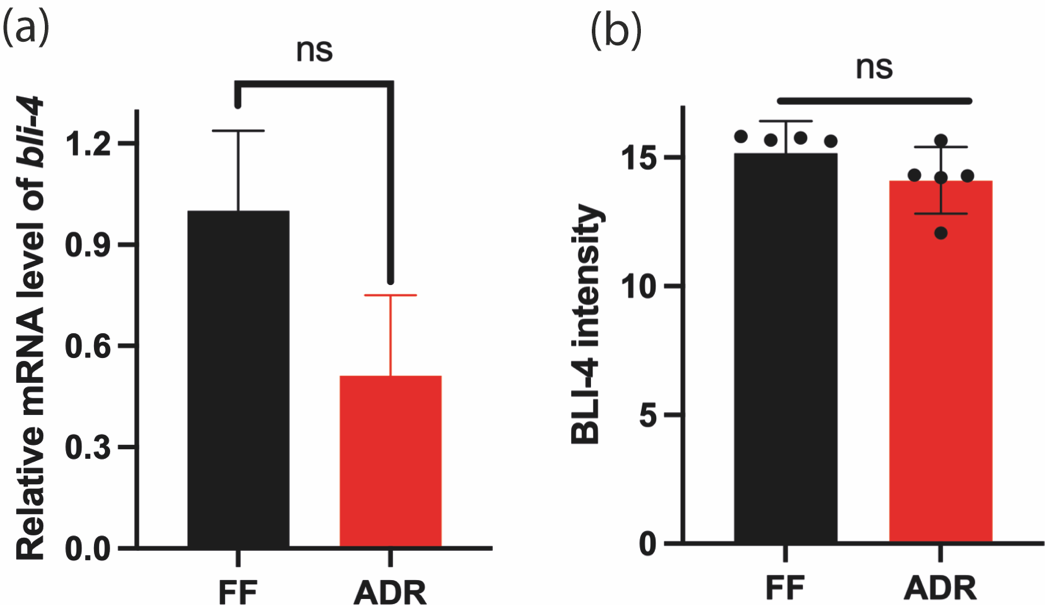


Figure S2. Expression levels of *bli-4* in FF and ADR conditions. (a) Relative mRNA levels of *bli-4* in FF and ADR conditions, measured by qRT-PCR. No significant difference (ns) was observed between the two dietary conditions. Data are presented as mean ± SEM. (b) BLI-4 protein intensity in FF and ADR conditions, also showing no significant difference (ns). Each dot represents an individual sample, with error bars indicating the SEM.


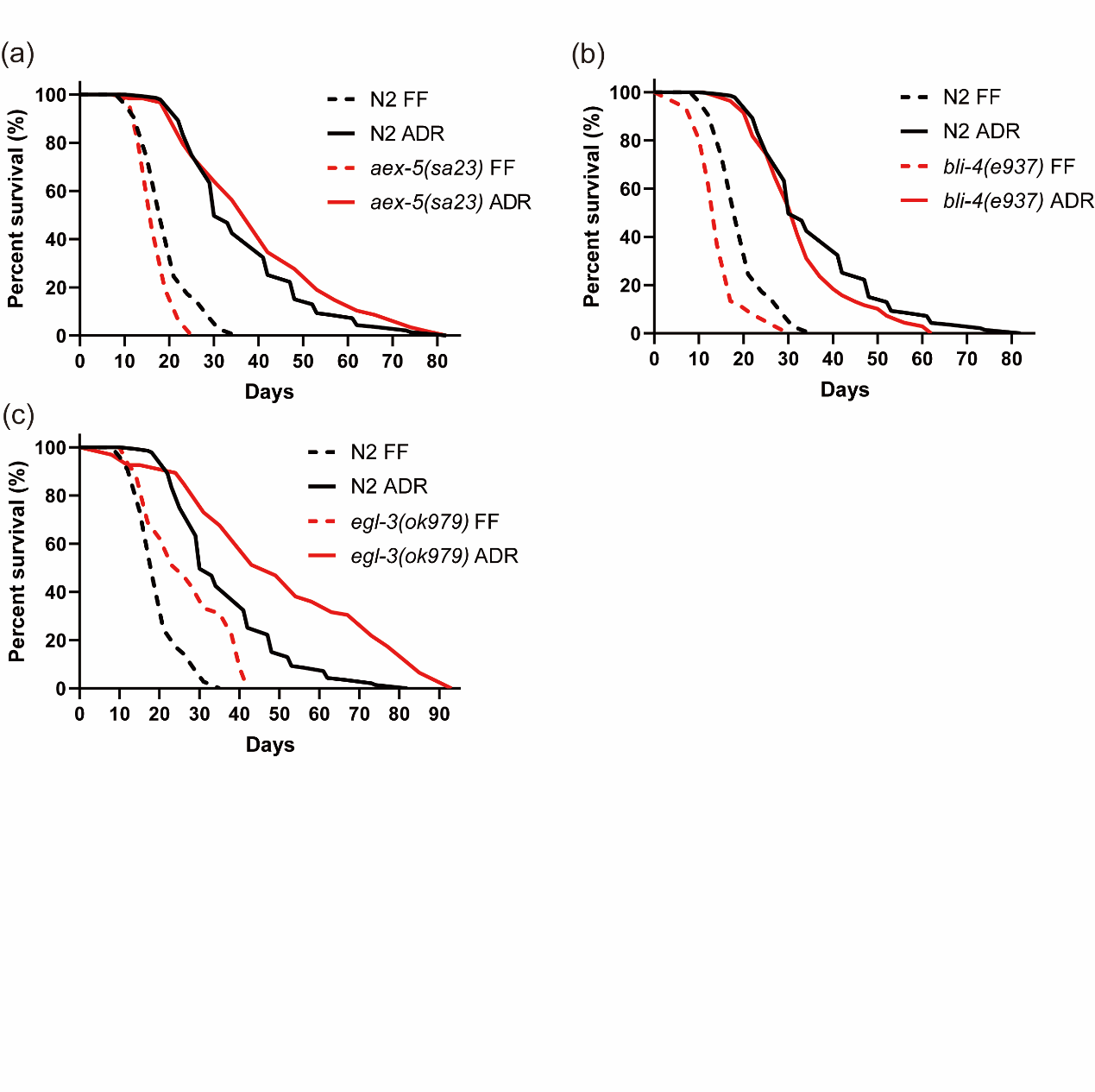


Figure S3. Survival of major proprotein convertase mutants (a) *aex-5(sa23)* mutant, (b) *bli-4(e937)* mutant, and (c) *egl-3(ok979)* mutant. FF=fully fed, ADR=axenic dietary restriction. *P-*values were calculated by log-rank tests with Bonferroni correction. See survival statistics in Supplementary Table 3&4.


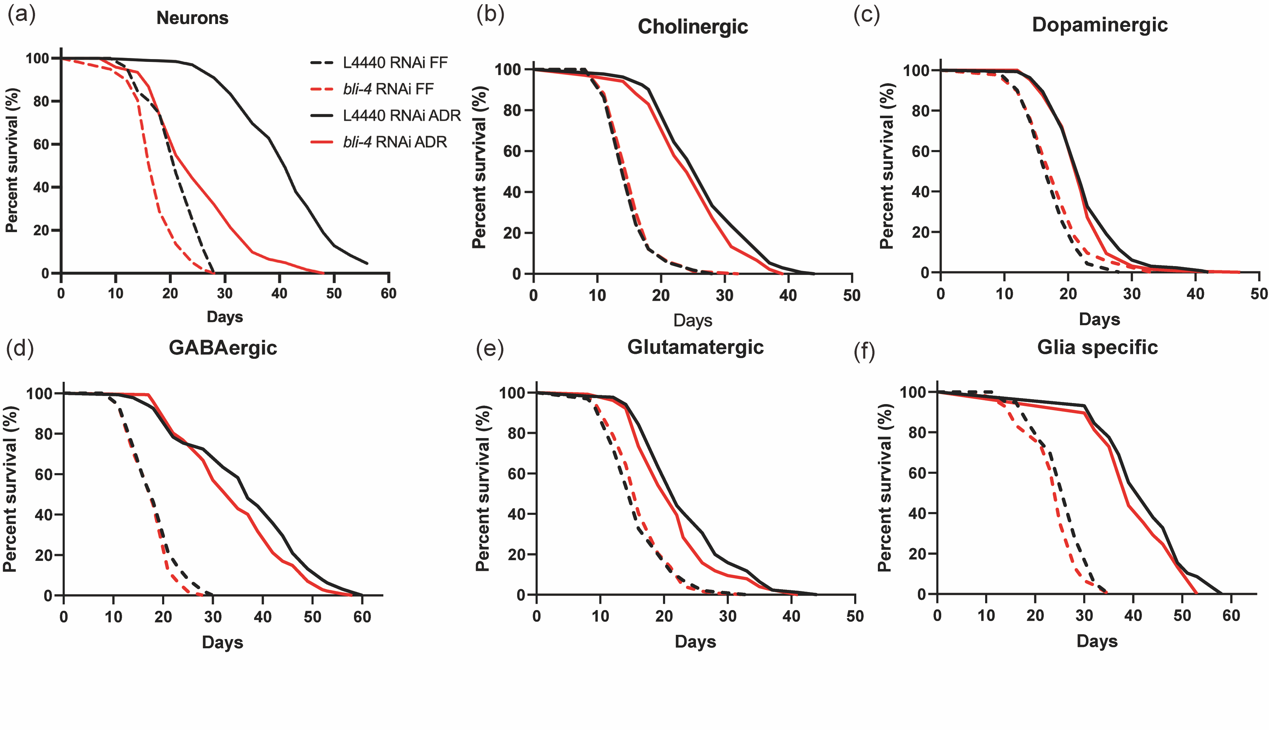


Figure S4. Effect of *bli-4* knockdown in (a) all neurons (AGD638 strain), (b) cholinergic neurons, (c) dopaminergic neurons, (d) GABAergic neurons, (e) glutamatergic neurons, and (f) amphid and phasmid sheath glia (Am/PH sheath cells) on survival under FF and ADR conditions. FF = fully fed, ADR = axenic dietary restriction. *P-*values were calculated by log-rank tests with Bonferroni correction. See survival statistics in Supplementary Table 3&4.

Figure S5. Relative contribution of tested genes for lifespan extension in axenic dilutions. Lifespan data and statistics for these tested genes are provided in Supplementary Tables 3&4. The contribution of specific genes to ADR lifespan extension was calculated as $Relative importance=\left( \frac{{N2}_{ADR}}{{N2}_{FF}}-\frac{{Mut}_{ADR}}{{Mut}_{FF}} \right)$, where N2_FF_ and N2_ADR_ represent the mean lifespan of wildtype worms under FF and specific ADR respectively, while Mut_FF_ and Mut_ADR_ denote the mean lifespan of mutant worms under these same conditions.


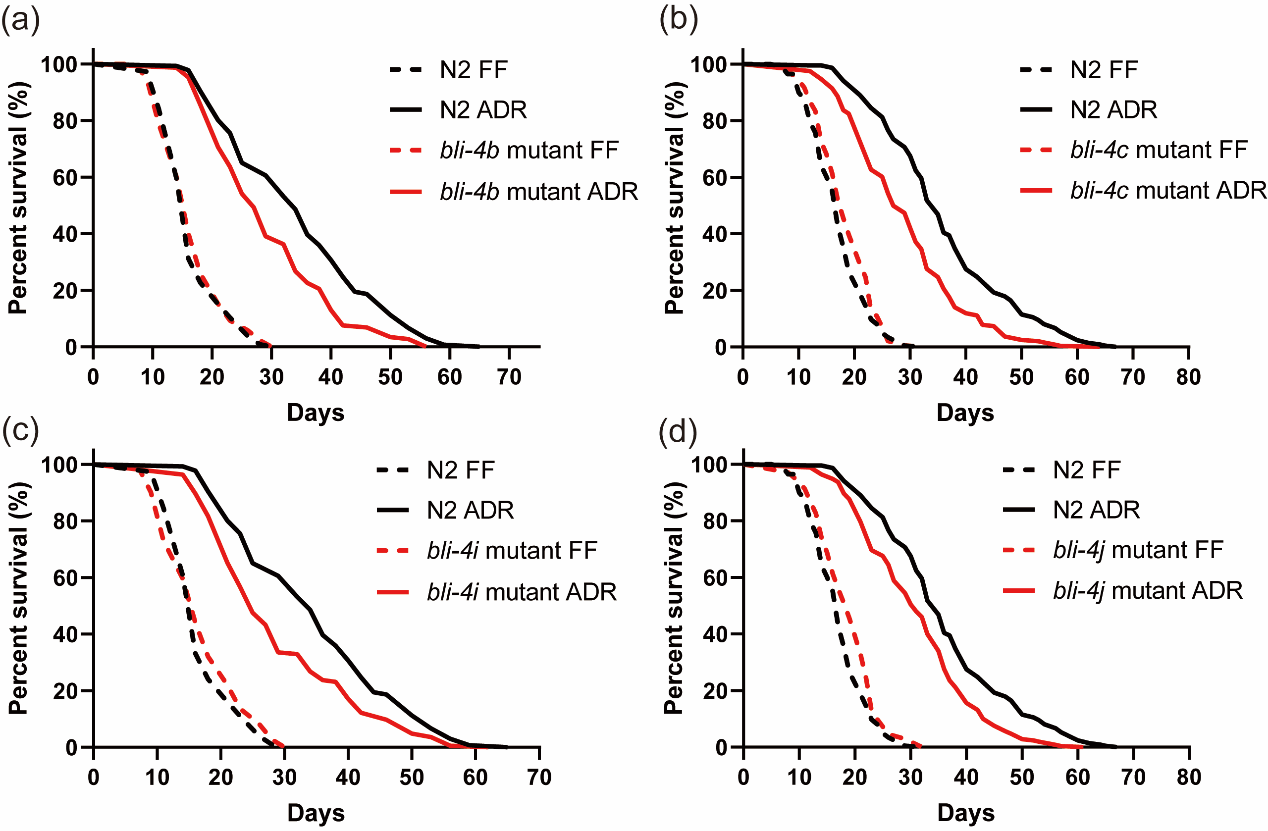


Figure S6. Survival of four specific isoforms mutants. (a) *bli-4b*, (b) *bli-4c*, (c) *bli-4i*, and (d) *bli-4j* isoforms mutants. FF = fully fed, ADR = axenic dietary restriction. As the *bli-4d* isoform is indispensable for development, generation of a mutant specific to this isoform is precluded. Furthermore, we were not able to attain viable homozygous *bli-4f* isoform mutants, also suggesting a developmental role for this isoform. *P-*values were calculated by log-rank tests with Bonferroni correction. See survival statistics in Supplementary Table 3&4.


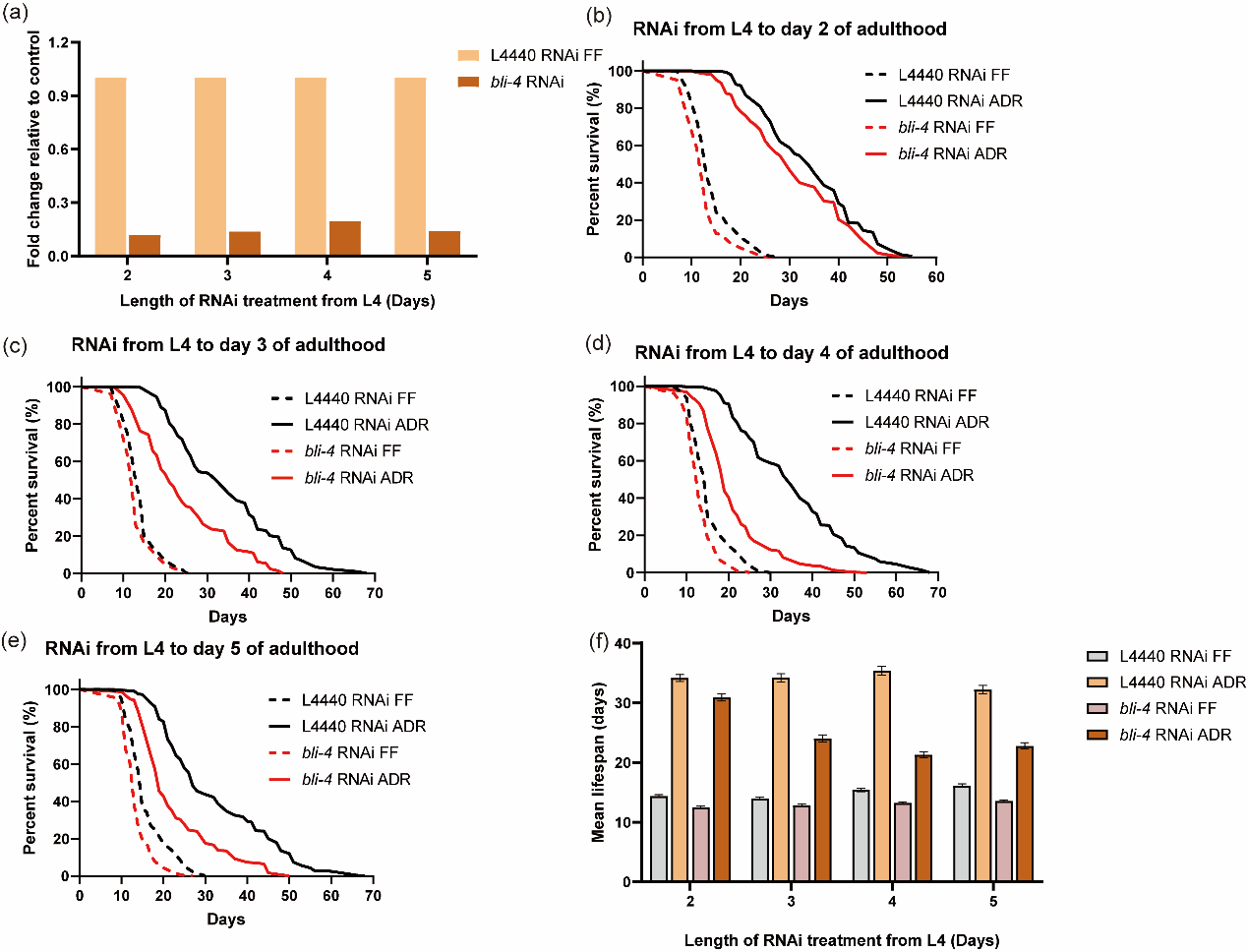


Figure S7. Three-day RNAi exposure was selected to obtain young animals for proteomics analysis. (a) quantification of *bli-4* by qPCR at various RNAi exposure periods. (b-e) Worm survival after RNAi treatment from L4 stage for different time periods: (b) 2 days, (c) 3 days, (d) 4 days and (e) 5 days in FF and ADR conditions. (f) mean lifespan of worms exposed to RNAi for different periods of time. FF = fully fed, ADR = axenic dietary restriction. *P-*values were calculated by log-rank tests with Bonferroni correction. See survival statistics in Supplementary Table 3&4.


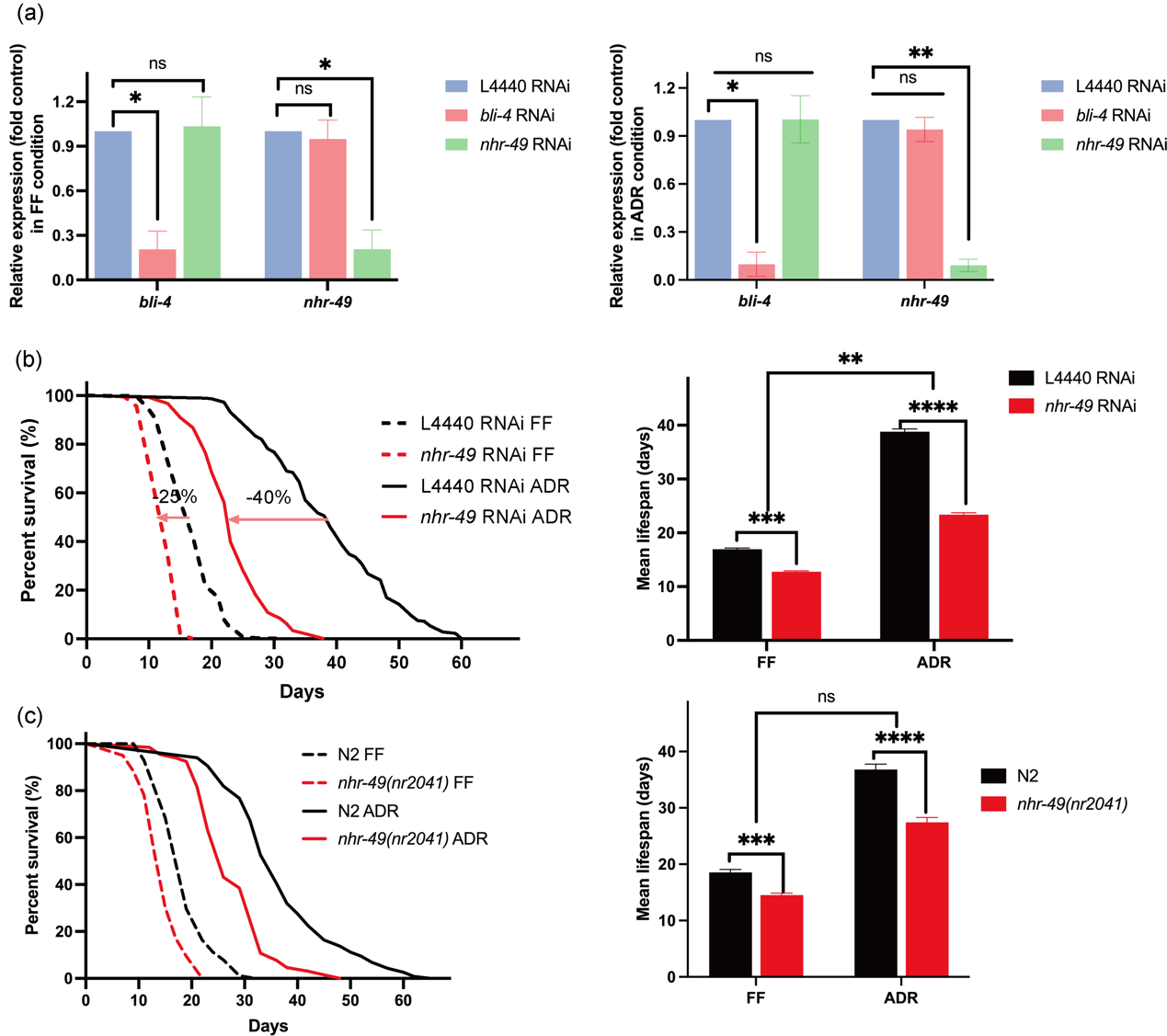


Figure S8. NHR-49 influences ADR-mediated longevity but may function independently of BLI-4. (a) Relative expression levels of *bli-4* and *nhr-49* under different RNAi conditions in FF (left panel) and ADR (right panel) states. Error bars represent mean ± SEM; statistical analysis by one-way ANOVA (n=2) (**P*<0.05, ***P*<0.01, ns, not significant). The effect of *nhr-49* knockdown (b) and *nhr-49(nr2041)* (c) on worm survival in ADR and FF conditions. FF = fully fed, ADR = axenic dietary restriction. *P-*values were calculated by log-rank tests with Bonferroni correction. See survival statistics in Supplementary Table 3&4.
